# Epigenetically silencing gluconeogenic enzyme PCK1 by EZH2 promotes renal tubulointerstitial fibrosis

**DOI:** 10.1186/s43556-026-00617-y

**Published:** 2026-10-14

**Authors:** Ming Wu, Pinglan Lin, Zhiqiang Yan, Yanzhe Wang, Anna Rinaldi, Pietro E. Cippà, Di Huang, Jiayi Qiao, Aidong Yang, Bo Tan, Chaoyang Ye, Dongping Chen

**Affiliations:** 1 Department of Nephrology, Shuguang Hospital Affiliated to Shanghai University of Traditional Chinese Medicine, Shanghai, China; 2 TCM Institute of Kidney Disease of Shanghai University of Traditional Chinese Medicine, Shanghai, China; 3 Key Laboratory of Liver and Kidney Diseases, Ministry of Education, Shanghai Key Laboratory of Traditional Chinese Clinical Medicine, Shanghai, China; 4 Clinical Pharmacokinetic Laboratory, Shuguang Hospital Affiliated to Shanghai University of Traditional Chinese Medicine, Shanghai, China; 5 Department of Medicine, Division of Nephrology, Ente Ospedaliero Cantonale, Lugano, Switzerland; 6 Laboratories for Translational Research, Ente Ospedaliero Cantonale, Bellinzona, Switzerland; 7 The Research Center for Traditional Chinese Medicine, Shanghai Institute of Infectious Diseases and Biosecurity, School of Traditional Chinese Medicine, Shanghai University of Traditional Chinese Medicine, Shanghai, China

**Keywords:** EZH2, Epigenetic regulation, Renal fibrosis, PCK1, Gluconeogenesis

## Abstract

Chronic kidney disease (CKD) is a leading cause of global morbidity. The kidney is a major gluconeogenic organ, and impaired renal gluconeogenesis contributes to CKD, yet the regulatory mechanisms remain poorly understood. Here we show that enhancer of zeste homolog 2 (EZH2), a histone H3K27 trimethyltransferase, epigenetically suppresses renal gluconeogenesis and drives tubulointerstitial fibrosis. Through integrative multi-omics profiling (RNA-seq, CUT&Tag, and single-nucleus RNA-seq), we show that EZH2 directly binds to and deposits H3K27 trimethylation at promoters of gluconeogenic genes in proximal tubular cells, with the most prominent repression observed at phosphoenolpyruvate carboxykinase (*Pck1*) gene. In fibrotic kidneys, *Ezh2* is markedly upregulated in proximal tubules and inversely correlates with *Pck1* expression across injury and maladaptive repair states. Genetic ablation or pharmacological inhibition of EZH2 restores PCK1 expression, enhances lactate-to-glucose conversion, and attenuates extracellular matrix deposition and fibrotic marker expression in both cultured tubular cells and mouse models of kidney fibrosis. Stable isotope ^13^C-lactate tracing shows that tubular *Ezh2* deletion increases the labelling fractions of gluconeogenic intermediates while reducing the labelling fraction of acetyl-CoA, indicating preferential partitioning of lactate-derived carbon towards gluconeogenesis rather than oxidative entry into the tricarboxylic acid (TCA) cycle in vivo. Mechanistically, *Pck1* gain- and loss-of-function experiments establish PCK1 as a non-redundant downstream effector of EZH2’s pro-fibrotic action. Collectively, our findings uncover an EZH2–PCK1 epigenetic–metabolic axis that couples histone methylation to gluconeogenic reprogramming and fibrogenesis. This axis may offer a dual therapeutic opportunity for CKD by concurrently restoring metabolic homeostasis and limiting fibrotic progression.

**Supplementary Information:**

The online version contains supplementary material available at https://doi.org/10.1186/s43556-026-00617-y.

## Introduction

Chronic kidney disease (CKD) affects nearly one in ten individuals globally, placing considerable strain on healthcare systems across the world [1, 2]. Despite advances in supportive care, the incidence of CKD continues to rise, and current therapeutic options remain limited in halting disease progression toward end-stage kidney disease [2]. The proximal tubule is particularly susceptible to renal injury, with tubulointerstitial fibrosis serving as a key predictor of renal survival [3, 4]. This process is accompanied by aberrant accumulation of extracellular matrix proteins (e.g., collagen I and fibronectin) and activation of epithelial–mesenchymal transition (EMT), as reflected by the upregulation of α-SMA, vimentin, and N-cadherin [3, 5]. Concomitantly, profibrotic signaling pathways, most notably the TGF-β pathway, are activated [6]. Recent studies have underscored the complex interplay between metabolic dysregulation and fibrotic progression in CKD, highlighting the need to elucidate the molecular drivers that link these two pathological features [7].

Beyond its traditional excretory functions, the kidney plays a crucial role in systemic energy regulation through gluconeogenesis, the metabolic process that synthesizes glucose from non-carbohydrate substrates including pyruvate and lactate [8]. This pathway involves 11 key enzymes, among which phosphoenolpyruvate carboxykinase 1 (PCK1), fructose-1,6-bisphosphatase 1 (FBP1), and glucose-6-phosphatase catalytic subunit (G6PC) are recognized as the primary rate-controlling steps [9]. Renal gluconeogenesis, predominantly occurring in the proximal tubules, plays a vital role in maintaining systemic glucose homeostasis during fasting [9]. Notably, emerging research indicates a significant interplay between renal gluconeogenesis and CKD progression [10]. Reduced gluconeogenic capacity has been observed in fibrotic mouse kidneys and correlates with poorer prognosis in CKD patients, underscoring its pathogenic significance [10]. However, the molecular mechanisms regulating impaired renal gluconeogenesis in CKD, particularly whether epigenetic dysregulation contributes to this defect, remain largely unclear.

Epigenetic regulation through histone modifications represents a dynamic layer of gene expression control that can significantly influence disease pathogenesis and progression [11]. Among these mechanisms, histone methylation plays a particularly important role in modulating chromatin accessibility and transcriptional activity [12]. Enhancer of zeste homolog 2 (EZH2), the catalytic subunit of polycomb repressive complex 2 (PRC2), functions as a histone methyltransferase by depositing trimethyl marks on lysine 27 of histone H3 (H3K27me3), thereby silencing its target genes at the transcriptional level [11, 13]. EZH2 has been implicated in numerous pathological processes, including cancer progression, immune dysregulation, and fibrotic diseases [11, 14, 15]. Studies have demonstrated that targeting EZH2 protects against acute kidney injury (AKI) induced by ischemia–reperfusion or folic acid, attenuates renal fibrosis in unilateral ureteral obstruction (UUO) models, and ameliorates crystal-induced kidney fibrosis [16–18]. Recent studies have further highlighted EZH2’s involvement in hepatic and renal carcinogenesis through repression of FBP1, a key gluconeogenic enzyme [19]. However, whether EZH2 regulates the broader gluconeogenic program in non-malignant kidney disease, particularly in the context of CKD progression, has remained unexplored.

Based on these observations, it was hypothesized that EZH2-mediated epigenetic repression of gluconeogenic genes contributes to the metabolic dysfunction and fibrotic progression observed in CKD. To test this hypothesis, an integrated multi-omics approach combining RNA-seq, CUT&Tag profiling, single-nucleus RNA-seq, and metabolic flux analysis were employed, together with genetic and pharmacological manipulation of EZH2 in both in vitro and in vivo models of renal fibrosis, including UUO and folic acid nephropathy. These results establish that EZH2 directly represses PCK1 and other gluconeogenic genes through H3K27me3 enrichment at their promoters in proximal tubules. This transcriptional repression compromises physiological renal gluconeogenic capacity, identifying the EZH2–PCK1 pathway as a previously unrecognized therapeutic target for chronic kidney disease.

## Results

### EZH2 promotes CKD progression

**Fig. 1:**
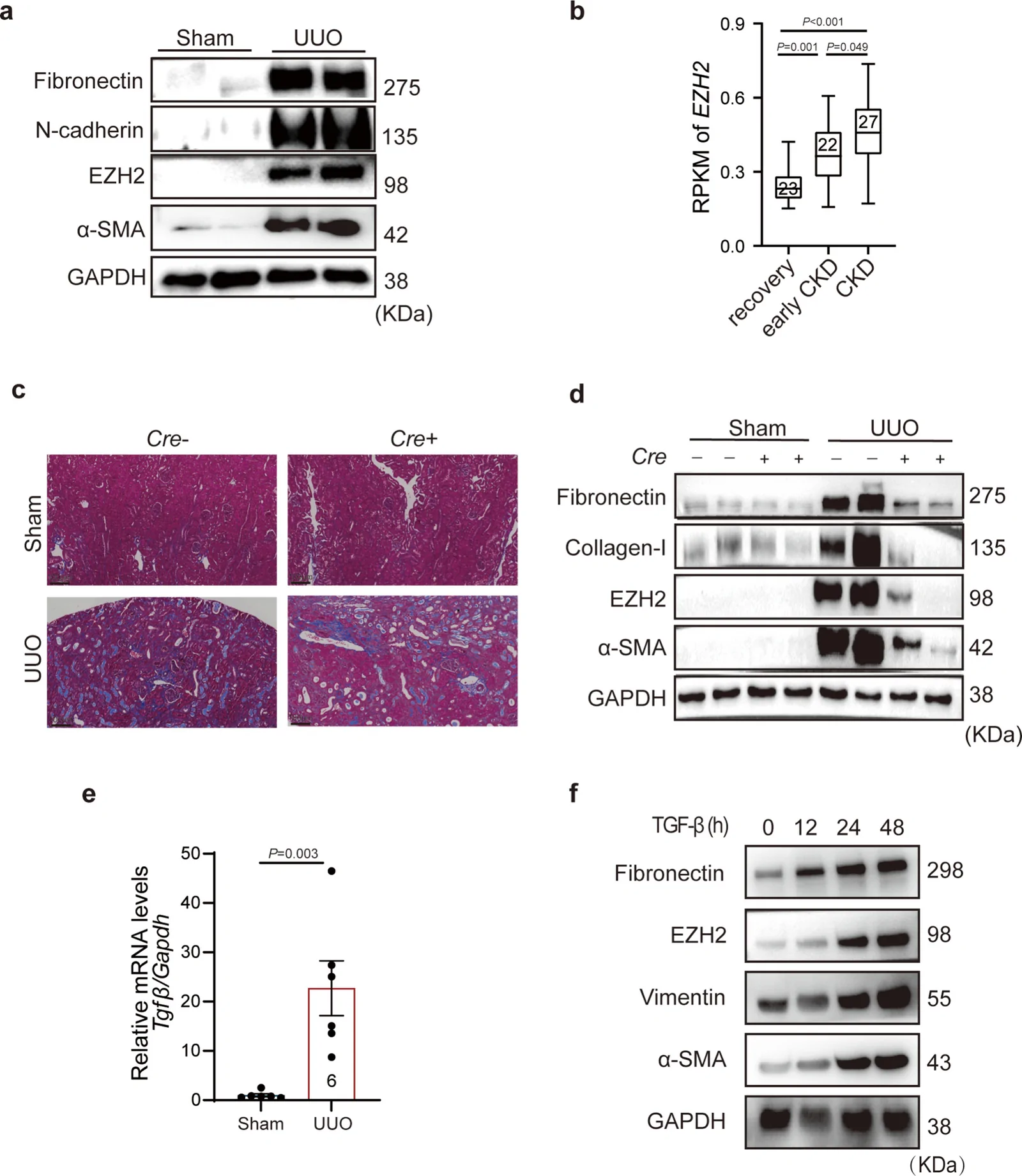
EZH2 is pro-fibrotic in injured kidneys and positively associated with human CKD progression. **a** Male wild type (WT) mice underwent sham surgery or unilateral ureteral obstruction (UUO) and were sacrificed at 14 days post-operation (*n* = 7 mice per group). Western blot analysis of fibronectin, N-cadherin, EZH2, and α-SMA expression in kidney tissues. *n* = 6 per group. **b** Secondary analysis of the publicly available human kidney allograft transcriptomic dataset (GEO: GSE126805). Kidney allograft biopsies at 3 and 12 months post-transplantation were categorized into three groups based on transcriptomic profiles: recovery (*n* = 23), early transition to CKD (*n* = 22), and CKD (*n* = 27). Box plots display *EZH2* RPKM values across groups with *p*-value from Kruskal–Wallis test. **c** Renal fibrosis evaluation by Masson’s trichrome staining in control (*Ezh2*^*fl/fl*^*;Cre/Esr1*^*−*^) and *Ezh2*-deleted (*Ezh2*^*fl/fl*^*;Cre/Esr1*^+^) male mice after sham or UUO surgery. Scale bar: 100 μm. *n* = 6 per group. **d** Western blot analysis of fibronectin, collagen-I, α-SMA, and EZH2 expression in renal tissues. *n* = 6 per group. **e** Male WT mice underwent sham surgery or UUO and were sacrificed at 14 days post-operation (*n* = 6 mice per group). qPCR analysis of *Tgf-β* in sham or UUO kidneys. **f** Western blot analysis of fibronectin, vimentin, EZH2, and α‑SMA in HK-2 cells stimulated with TGF-β at different time points. *n* = 3 per group. All cell‑based experiments were repeated independently three times, with each data point representing an independent biological sample

To investigate the role of EZH2 in renal fibrosis, its expression was first examined in the unilateral ureteral obstruction (UUO) model, a well-established mouse model of progressive tubulointerstitial fibrosis. EZH2 expression was significantly elevated in fibrotic kidneys from the UUO model, which correlated with increased fibrotic markers including fibronectin, N-cadherin, and α-SMA (Fig. 1a and Fig. S1a). The clinical relevance of EZH2 in human CKD was evaluated by RNA-seq of kidney-allograft biopsies. Patients showing CKD progression displayed progressively higher *EZH2* expression than those who recovered (Fig. 1b), indicating that EZH2 upregulation is a conserved feature of progressive kidney disease in both mice and humans.

A causal role for EZH2 in renal fibrosis was demonstrated by genetic ablation of *Ezh2* using the tamoxifen-inducible CAG-Cre/Esr1 system (*Ezh2*^*fl/fl*^*;Cre/Esr1*^+^). Following UUO, *Ezh2*-deleted kidneys exhibited significantly less extracellular matrix deposition than control littermates (*Ezh2*^*fl/fl*^*;Cre/Esr1*^*−*^) as shown by Masson’s trichrome staining (Fig. 1c and Fig. S1b). Efficient *Ezh2* deletion also lowered expression of pro-fibrotic markers fibronectin, collagen-I, and α-SMA, as confirmed by Western blotting (Fig. 1d and Fig. S1c). These findings suggest that EZH2 is a critical pro-fibrotic factor in CKD pathogenesis.

Whether the TGF‑β signaling pathway acts upstream of EZH2 in fibrotic kidneys was next investigated. As shown in Fig. 1e, *Tgf-β* mRNA levels were upregulated in mouse kidneys following UUO surgery. Stimulation of HK-2 renal epithelial cells with TGF-β increased EZH2 expression in a time-dependent manner, accompanied by upregulation of pro-fibrotic markers including fibronectin, vimentin, and α-SMA (Fig. 1f and Fig. S2).

### Multi-omics analysis reveals a negative correlation between EZH2 expression and renal gluconeogenesis in fibrotic kidneys

**Fig. 2:**
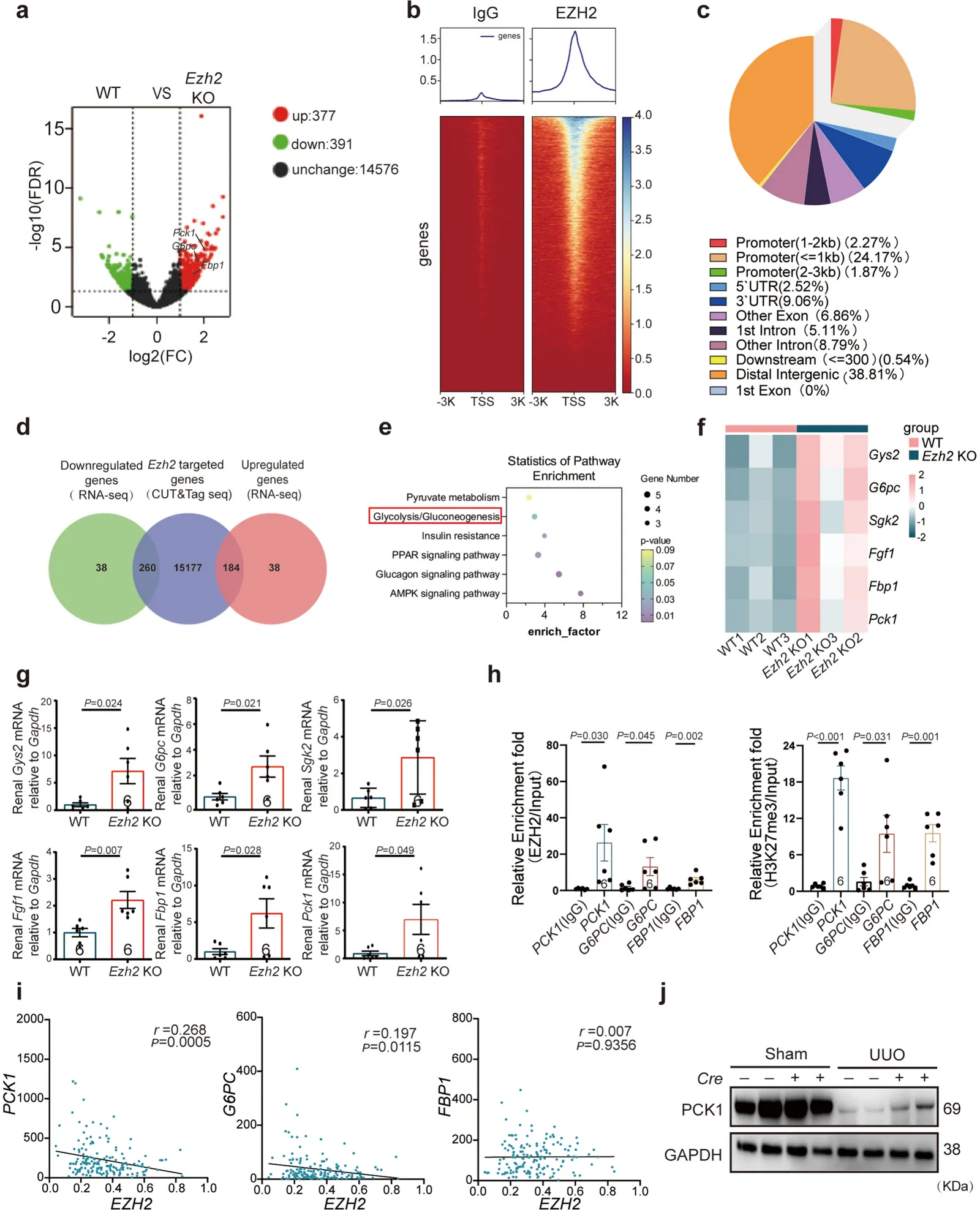
EZH2 negatively regulates renal gluconeogenesis in CKD. **a** Volcano plots of differentially expressed genes between wild-type control (*Ezh2*^*fl/fl*^*;Cre/Esr1*^*−*^) (referred to WT) and *Ezh2*-deleted (*Ezh2*^*fl/fl*^*;Cre/Esr1*^+^) (referred to *Ezh2*_KO) UUO kidneys by RNA-seq. **b** CUT&Tag sequencing enrichment profiles for IgG and EZH2 across ± 3 kb of transcription start sites (TSS) in TGF-β-stimulated HK-2 cells. Upper panels: average enrichment profiles; lower panels: read-density heatmaps. **c** Genomic annotation of EZH2 binding peaks from CUT&Tag sequencing in TGF-β-stimulated HK-2 cells. **d** Venn diagram illustrating overlap between EZH2-regulated genes from RNA-seq and EZH2-binding genes from CUT&Tag sequencing. **e** KEGG pathway enrichment analysis of EZH2-targeted genes. **f** Heatmap of expression values for genes enriched in EZH2-regulated pathways (red: up-regulation, green: down-regulation). **g** qPCR analysis of EZH2-targeted genes in control versus *Ezh2* conditional knockout UUO kidneys. **h** CUT&Tag-qPCR showing EZH2 and H3K27me3 occupancy on *PCK1, FBP1* and *G6PC* promoters in TGF-β-stimulated HK-2 cells, with IgG as the negative control. *n* = 6 per group. **i** Pearson’s correlation analysis between *EZH2* and gluconeogenesis-related genes in kidney allograft biopsies from the publicly available dataset (GEO: GSE126805) (*n* = 163). **j** Western blot analysis of PCK1 expression in control (*Ezh2*^*fl/fl*^*;Cre/Esr1*^*−*^) and *Ezh2*-deleted (*Ezh2*^*fl/fl*^*;Cre/Esr1*^+^) male mice with sham or UUO operation. *n* = 6 per group. Comparisons among multiple groups were performed using one-way ANOVA followed by Tukey’s post-hoc test, while comparisons between two groups were conducted using unpaired two-tailed Student’s *t*‑tests. Data are presented as mean ± standard deviation

To dissect the mechanisms underlying EZH2’s pro-fibrotic effects, RNA-seq was performed on *Ezh2* knockout and control UUO kidneys. A total of 768 differentially expressed genes (DEGs) were identified: 391 were downregulated and 377 were upregulated (Log_2_FC ≥ 1, FDR ≤ 0.05) in *Ezh2*-deficient kidneys (Fig. 2a). Direct EZH2 targets were mapped by CUT&Tag-Seq in TGF-β-treated HK-2 cells (a well-established in vitro model of renal fibrosis), yielding 15,177 EZH2 binding peaks, 28.31% of which were located within promoter regions (≤ 3 kb from the transcription start sites) (Fig. 2b, c).

Integration of the two datasets revealed 184 genes that were both upregulated in *Ezh2* knockout UUO kidneys and occupied by EZH2, consistent with its role as a transcriptional repressor (Fig. 2d). KEGG analysis showed significant enrichment in metabolic pathways, including gluconeogenesis (Fig. 2e). A heatmap confirmed marked upregulation of gluconeogenic genes in *Ezh2*-deficient kidneys (Fig. 2f). qPCR validated higher mRNA levels of the key gluconeogenic genes *Pck1*, *Fbp1*, and *G6pc* in UUO kidneys after *Ezh2* deletion (Fig. 2g).

CUT&Tag-qPCR in TGF-β-stimulated HK-2 cells demonstrated significant enrichment of both EZH2 and its associated repressive histone mark H3K27me3 at the promoters of *PCK1*, *FBP1*, and *G6PC*, with the greatest enrichment observed at the *PCK1* promoter (Fig. 2h). Analysis of the human kidney allograft dataset further indicated a significant negative correlation between *EZH2* and *PCK1* or *G6PC* expression, whereas no clear correlation was found between *EZH2* and *FBP1* expression (Fig. 2i). Western blotting confirmed markedly higher PCK1 protein levels in *Ezh2*-deficient UUO kidneys (Fig. 2j and Fig. S1c). Comparable results were obtained in a folic acid (FA) nephropathy model, where *Ezh2* deletion reduced EZH2 expression and increased PCK1 levels while reducing extracellular matrix accumulation and pro-fibrotic marker (fibronectin, collagen-I, and α-SMA) expression (Fig. S3a-e). Collectively, these findings suggest that gluconeogenic genes, particularly *PCK1*, are direct epigenetic targets of EZH2 in the context of renal fibrosis.

### Tubular EZH2 inhibits renal gluconeogenesis in models of kidney fibrosis

**Fig. 3:**
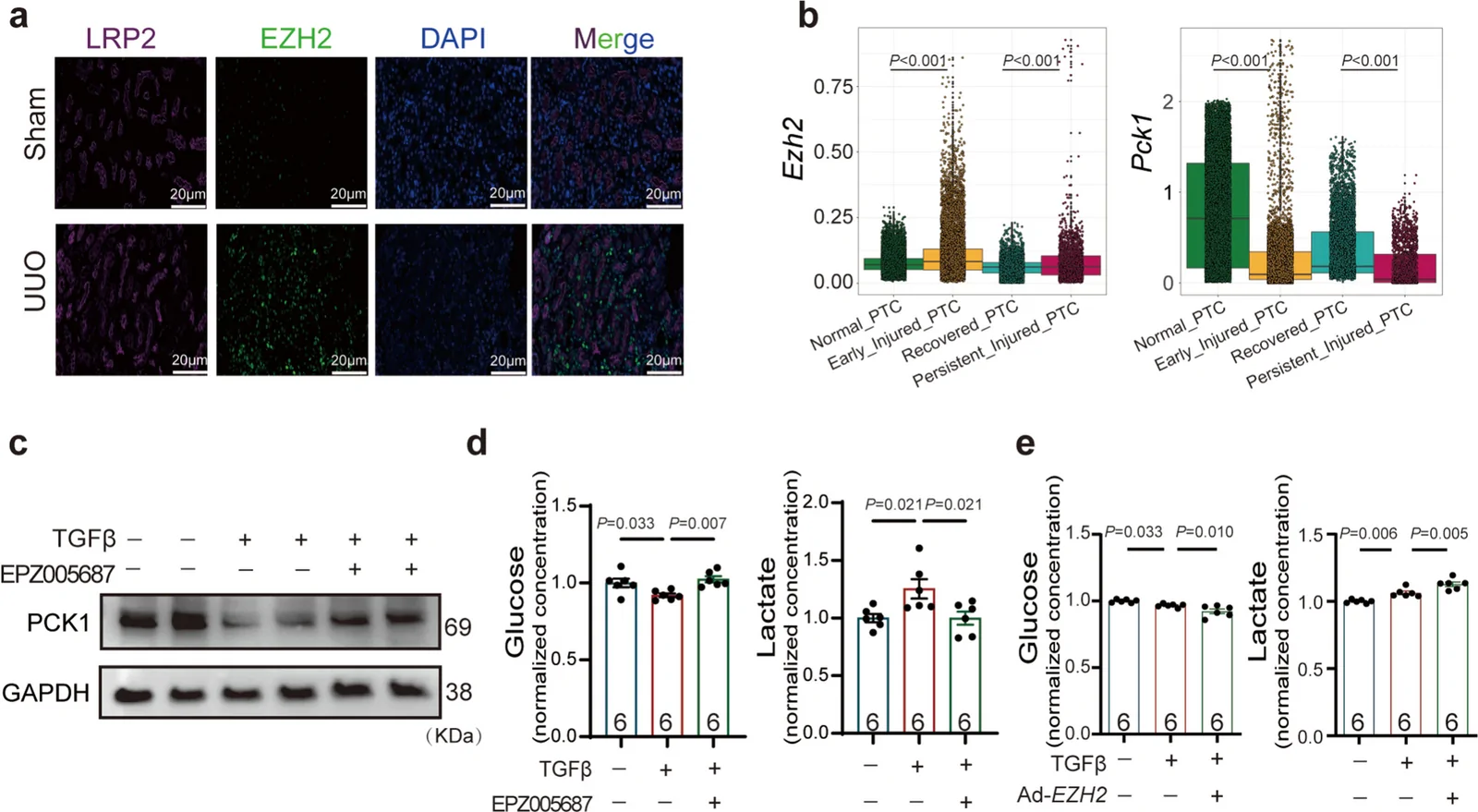
Tubular EZH2 inhibits renal gluconeogenesis. **a** Immunofluorescence staining for EZH2 and proximal tubular marker LRP2 in sham or UUO kidneys. Nuclei were counterstained with DAPI. Scale bars: 20 µm. *n* = 6 per group. **b** snRNA-seq analysis of *Pck1* and *Ezh2* expression in proximal tubule (PT) cells after bilateral ischemia–reperfusion injury (IRI) was used to investigate the expression of *Pck1* and *Ezh2* in uninjured and injured proximal tubule (PT) cells in the early and late phases after injury. Four PT cell states: Normal PT cells (positive for *Lrp2*, *Slc5A2*, *Slc13a3*, and *Slc16a9* before injury, green), Early injured PT cells (*Havcr1* positive at 4, 12, and 48 h after IRI, yellow), Recovered PT cells (positive for *Lrp2*, *Slc5A2*, *Slc13a3*, and *Slc16a9* at 14 days and 6 weeks, light blue), Persistently injured PT cells (positive for *Havcr1* and *Vcam1* at 14 days and 6 weeks, red). Box plots display MAGIC-imputed RNA expression (*Ezh2*: left panel; *Pck1*: right panel) with significance by Wilcoxon rank-sum test (FDR-adjusted). **c** Western blot analysis of PCK1 in HK-2 cells after TGF-β stimulation in the presence or absence of EPZ005687 (5 µM) for 48 h. *n* = 6 per group. **d** Glucose and lactate concentrations in the medium were measured after HK‑2 cells were treated with or without EPZ005687 (5 µM) for 48 h, and normalized to the control group (without TGF‑β stimulation). *n* = 6 per group. **e** Following transfection with adenovirus encoding *EZH2* (Ad‑*EZH2*) or control virus and stimulation with TGF‑β for 48 h, glucose and lactate concentrations in the medium of HK‑2 cells were measured and normalized to the control group (without TGF‑β stimulation). *n* = 6 per group. Comparisons among multiple groups were performed using one-way ANOVA followed by Tukey’s post-hoc test. Data are presented as mean ± standard deviation

Given that renal gluconeogenesis occurs predominantly in proximal tubular cells [8], EZH2 expression was next examined in this cell population. Immunofluorescence co-staining for EZH2 with the proximal tubular marker LRP2/megalin revealed prominent, nuclear-enriched EZH2 in proximal tubules of fibrotic kidneys (Fig. 3a).

Single-nucleus RNA-seq (snRNA-seq) from bilateral renal ischemia–reperfusion injury (IRI) further revealed dynamic, reciprocal changes: injured proximal tubule cells showed higher *Ezh2* expression and lower *Pck1* expression, a pattern that persisted during maladaptive repair (Fig. 3b).

EPZ005687, a potent and highly selective inhibitor of EZH2 [20, 21], restored PCK1 expression (Fig. 3c), blunted lactate accumulation, and raised glucose concentration (Fig. 3d) in TGF-β-stimulated HK-2 cells, while reducing the expression of pro-fibrotic markers fibronectin and α-SMA (Fig. S4a, b). Conversely, adenoviral *EZH2* overexpression reduced glucose levels and increased lactate accumulation, accompanied by elevated fibronectin expression (Fig. 3e and Fig. S5a-b).

**Fig. 4:**
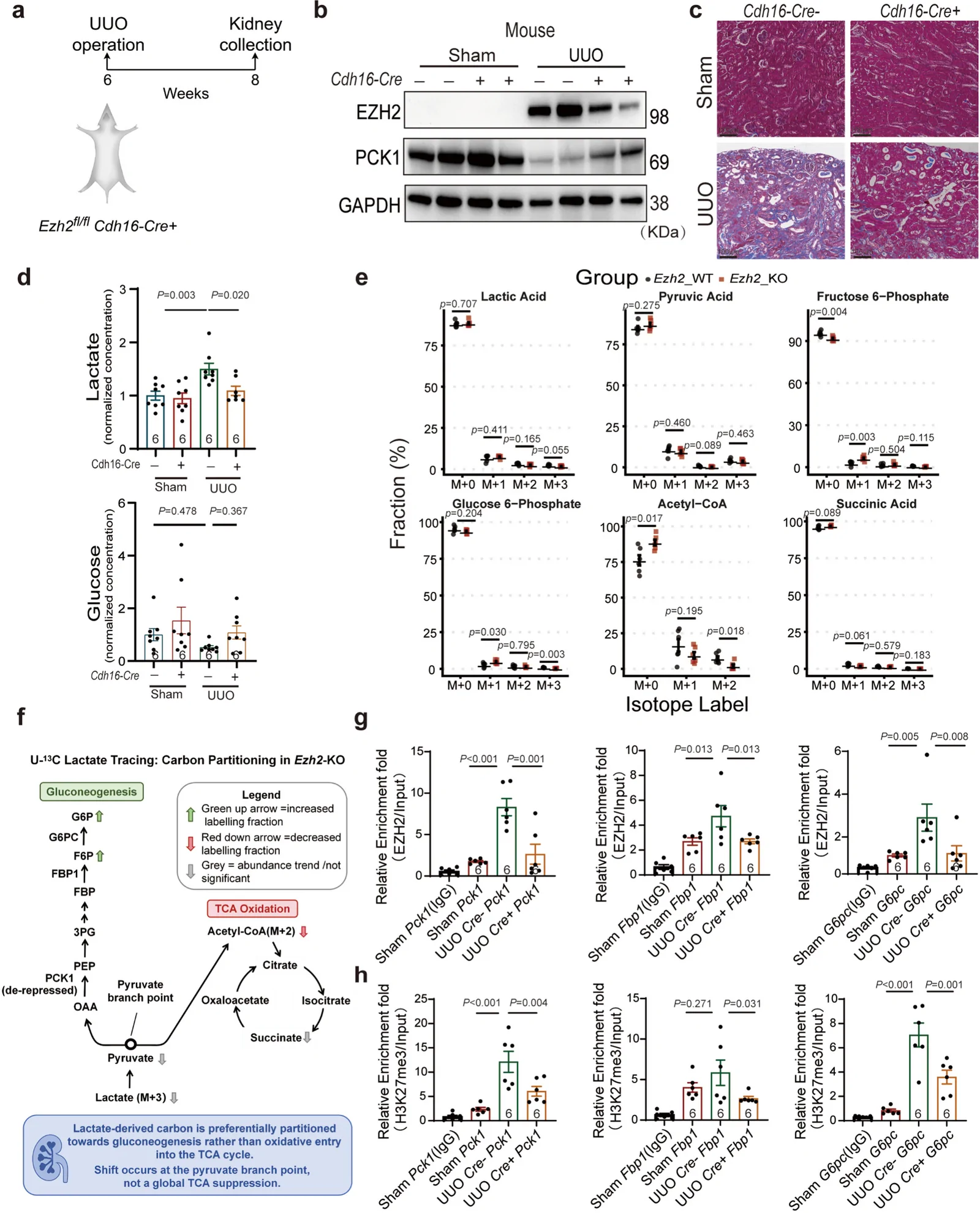
Deletion of *Ezh2* in renal tubules promotes renal gluconeogenesis. **a** Experimental design: male mice with or without tubular-specific deletion of *Ezh2* (*Ezh2*^fl/fl^;*Cdh16-Cre*) underwent sham or UUO surgery at 6 weeks of age and were sacrificed after 2 weeks (*n* = 7 mice per group). **b** Western blot analysis of PCK1 and EZH2 in kidney samples. *n* = 6 per group. **c** Masson’s trichrome staining of renal fibrosis. Scale bar: 100 μm. *n* = 6 per group. **d** Kidney lactate and glucose levels were measured and normalized to sham kidneys (without *Ezh2* deletion). *n* = 7 per group. **e** Male mice with tubular-specific deletion of *Ezh2* (*Ezh2*^fl/fl^;*Cdh16-Cre*) (referred to *Ezh2*_KO) or control (referred to *Ezh2*_WT) littermates at 6 weeks of age were subjected to UUO. After 16 h of fasting, mice received intraperitoneal injections of ^13^C-lactate (dissolved in normal saline) on day 7 at a total dose of 10 mmol/kg, administered in three divided doses at 15-min intervals. Renal cortex samples were collected 60 min after the first injection and subjected to metabolomic analysis of ^13^C enrichment. Fractional labeling of gluconeogenic intermediates is shown. *n* = 6 per group. **f** Schematic summary of the ^13^C-lactate tracing experiment in *Ezh2* (*Ezh2*^fl/fl^;*Cdh16-Cre*) knockout (KO) mice. Abbreviations: OAA, oxaloacetate; PEP, phosphoenolpyruvate; 3PG, 3-phosphoglycerate; FBP, fructose-1,6-bisphosphate; F6P, fructose 6-phosphate; G6P, glucose 6-phosphate; Acetyl-CoA, acetyl-coenzyme A; TCA cycle, tricarboxylic acid cycle; PCK1, phosphoenolpyruvate carboxykinase 1; FBP1, fructose-1,6-bisphosphatase 1; G6PC, glucose-6-phosphatase catalytic subunit. **g**-**h** Male mice with tubular-specific deletion of *Ezh2* (*Ezh2*^fl/fl^;*Cdh16-Cre*) underwent sham or UUO surgery at 6 weeks of age and were sacrificed after 2 weeks (*n* = 6 per group). Primary renal cells were isolated from mouse cortex. CUT&Tag qPCR showing EZH2 and H3K27me3 occupancy on *Pck1, Fbp1* and *G6pc* promoters in renal primary cells, with IgG as the negative control. Comparisons among multiple groups were performed using one-way ANOVA followed by Tukey’s post-hoc test. Data are presented as mean ± standard deviation

In vivo, tubular-specific *Ezh2* deletion (*Ezh2*^*fl/fl*^*;Cdh16-Cre*) prevented PCK1 downregulation, reduced the expression of fibrotic markers (fibronectin, N-cadherin, and α-SMA), and lessened matrix deposition after UUO (Fig. 4a-c and Fig. S6a-d). UUO-induced renal lactate accumulation was also diminished by tubular *Ezh2* deletion (Fig. 4d). While renal glucose levels decreased after UUO and exhibited a rising trend upon *Ezh2* deletion, no statistically significant differences were found among the three groups (Fig. 4d).

To obtain direct in vivo evidence, ^13^C-lactate tracing was performed in mice with or without tubular-specific *Ezh2* deletion following UUO. In *Ezh2*-deficient kidneys, the gluconeogenic intermediates fructose 6-phosphate (M + 1) and glucose 6-phosphate (M + 1) showed significantly increased labelling fractions, whereas the labelling fraction of acetyl-CoA (M + 2), which reports pyruvate-derived carbon entry into the tricarboxylic acid (TCA) cycle via pyruvate dehydrogenase, was significantly reduced (Fig. 4e, f). Labelling of downstream TCA cycle intermediates including succinate was not significantly altered, suggesting that the shift occurs primarily at the pyruvate branch point rather than reflecting a global suppression of TCA cycle activity (Fig. 4e, f). Consistent findings were obtained when isotopologue abundances were analyzed, with labelled lactate (M + 3, log_2_FC = − 0.555, *p* = 0.013) and acetyl-CoA (M + 2) showing significant reductions, whereas fructose 6-phosphate (M + 1, log_2_FC = 1.214, *p* = 0.0034) and glucose 6-phosphate (M + 1, log_2_FC = 0.858, *p* = 0.0296) were significantly increased (Fig. S6e). Together, these reciprocal changes indicate that tubular *Ezh2* deletion preferentially partitions lactate-derived carbon towards gluconeogenic intermediates rather than oxidative entry into the TCA cycle, in keeping with de-repression of PCK1.

To gain deeper mechanistic insight into EZH2-mediated epigenetic regulation of gluconeogenic genes in vivo, CUT&Tag-qPCR analysis was performed in primary kidney cells from sham or UUO kidneys. This revealed significant enrichment of both EZH2 and H3K27me3 at the promoters of *Pck1*, *Fbp1*, and *G6pc* in UUO kidneys compared with sham controls (Fig. 4g, h). Furthermore, tubular deletion of *Ezh2* reduced the enrichment of EZH2 and H3K27me3 at these promoter regions (Fig. 4g, h). Together, these findings indicate that tubular EZH2 suppresses renal gluconeogenesis in fibrotic kidneys.

### Renal gluconeogenesis protects against renal fibrosis

**Fig. 5:**
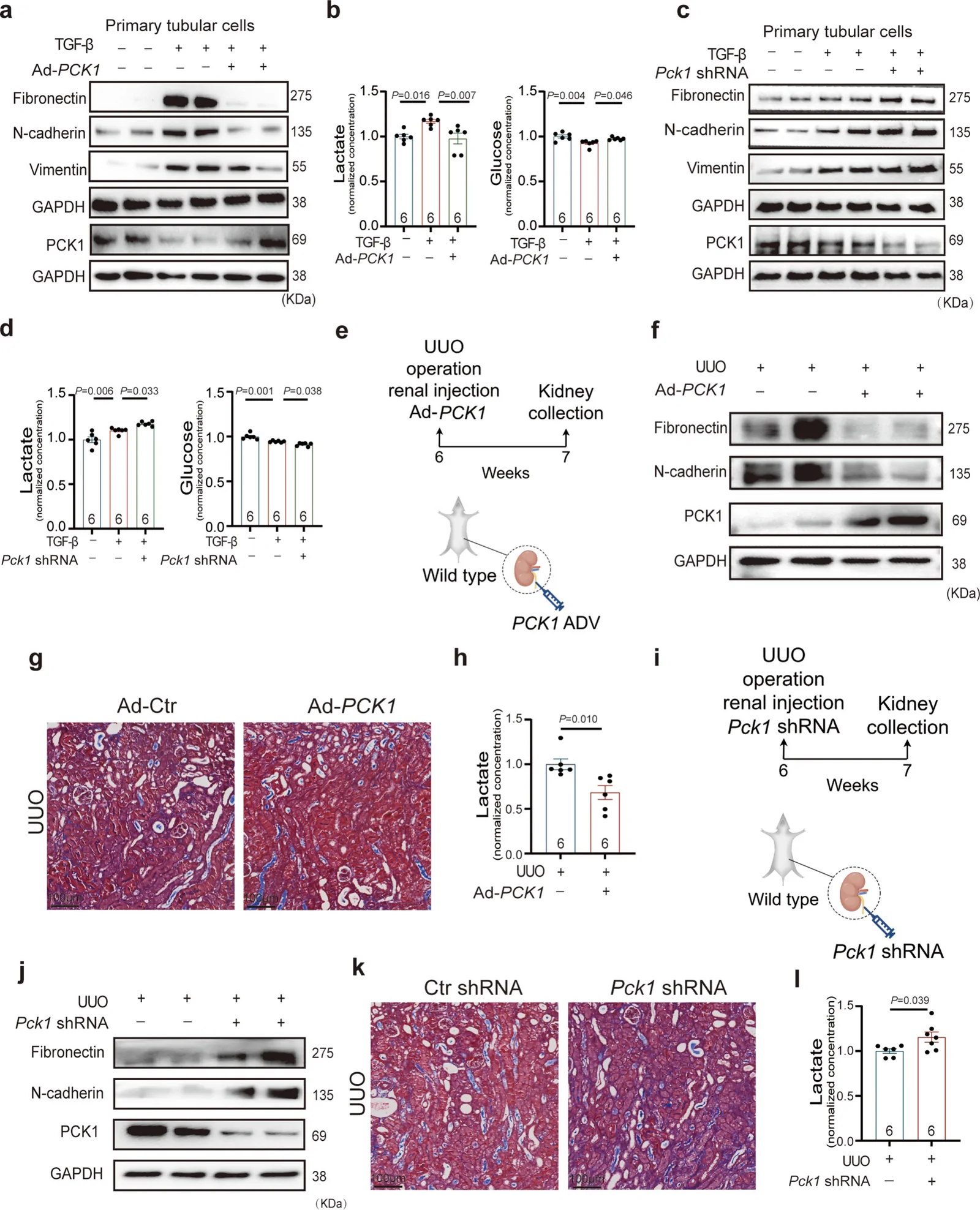
PCK1 inhibits renal fibrosis. **a** Western blot analysis of fibronectin, N-cadherin, vimentin, and PCK1 in primary tubular cells transfected with adenovirus encoding *PCK1* (Ad-*PCK1*) or control virus (Ad-Ctr) and stimulated with TGF-β for 48 h. *n* = 6 per group. **b** Glucose and lactate concentrations in cell medium were measured and normalized to the control group (without TGF‑β stimulation). *n* = 6 per group. **c** Western blot analysis of fibronectin, N-cadherin, vimentin, and PCK1 in primary tubular cells transfected with *Pck1* shRNA or control shRNA and stimulated with TGF-β for 48 h. *n* = 6 per group. **d** Glucose and lactate concentrations in cell medium from primary tubular cells transfected with *Pck1* shRNA or control shRNA and stimulated with TGF-β for 48 h were measured, and normalized to the control group (without TGF‑β stimulation). *n* = 6 per group. **e** Experimental design: Wild-type (WT) male mice injected with Ad-Ctr or Ad-*PCK1* underwent UUO surgery at 6 weeks of age and were sacrificed after 1 week (*n* = 6 per group). **f** Western blot analysis of fibronectin, N-cadherin, and PCK1. *n* = 6 per group. **g** Masson’s trichrome staining of renal fibrosis in UUO kidneys with control virus or Ad-*PCK1*. Scale bar: 100 µm. *n* = 6 per group. **h** Kidney lactate levels were measured and normalized to UUO kidneys without *PCK1* overexpression. *n* = 6 per group. **i** Experimental design: Wild-type (WT) male mice injected with control shRNA or *Pck1* shRNA underwent UUO surgery at 6 weeks of age and were sacrificed after 1 week (*n* = 6 per group). **j** Western blot analysis of fibronectin, N-cadherin, and PCK1. *n* = 6 per group. **k** Masson’s trichrome staining of renal fibrosis in UUO kidneys with control shRNA or *Pck1* shRNA. Scale bar: 100 μm. *n* = 6 per group. **l** Kidney lactate levels were measured and normalized to UUO kidneys without *Pck1* shRNA. *n* = 6 per group. Comparisons among multiple groups were performed using one-way ANOVA followed by Tukey’s post-hoc test, while comparisons between two groups were conducted using unpaired two-tailed Student’s *t*‑tests. Data are presented as mean ± standard deviation

The direct role of PCK1 in renal fibrosis was confirmed through gain- and loss-of-function experiments. *PCK1* overexpression in TGF-β-stimulated primary tubular cells (PTCs) significantly reduced fibronectin, N-cadherin, and vimentin expression (Fig. 5a and Fig. S7a), and enhanced glucose production while reducing lactate accumulation (Fig. 5b). Conversely, *Pck1* knockdown via shRNA increased fibrotic marker (fibronectin, N-cadherin, and vimentin) expression and produced the opposite metabolic profile (reduced glucose concentration and increased lactate concentration) (Fig. 5c, d and Fig. S7b).

In vivo, intra-renal delivery of *PCK1* adenovirus attenuated UUO fibrosis, as evidenced by reduced fibrotic marker (fibronectin and N-cadherin) expression and matrix deposition (Fig. 5e-g and Fig. S7c, d), as well as lower renal lactate levels (Fig. 5h). However, *PCK1* overexpression did not increase renal glucose concentrations (Fig. S7e). *Pck1* knockdown exacerbated fibrosis as shown by increased fibrotic marker (fibronectin and N-cadherin) expression and enhanced extracellular matrix deposition (Fig. 5i-k and Fig. S7f, g). Deletion of the *Pck1* gene increased lactate levels but did not affect renal glucose levels (Fig. 5l and Fig. S7h). These results indicate that PCK1-mediated gluconeogenesis protects against kidney fibrosis.

To further validate the role of renal gluconeogenesis in renal fibrosis, FBP1 was pharmacologically inhibited in TGF-β‑stimulated HK-2 cells using FBPase-1 inhibitor‑1. The inhibition of FBP1 potentiated TGF-β‑induced fibrotic responses, as evidenced by increased expression of fibronectin, N-cadherin, and vimentin, along with elevated lactate levels and decreased glucose concentrations (Fig. S8a-c).

### EZH2 promotes renal fibrosis through inhibition of gluconeogenesis

**Fig. 6:**
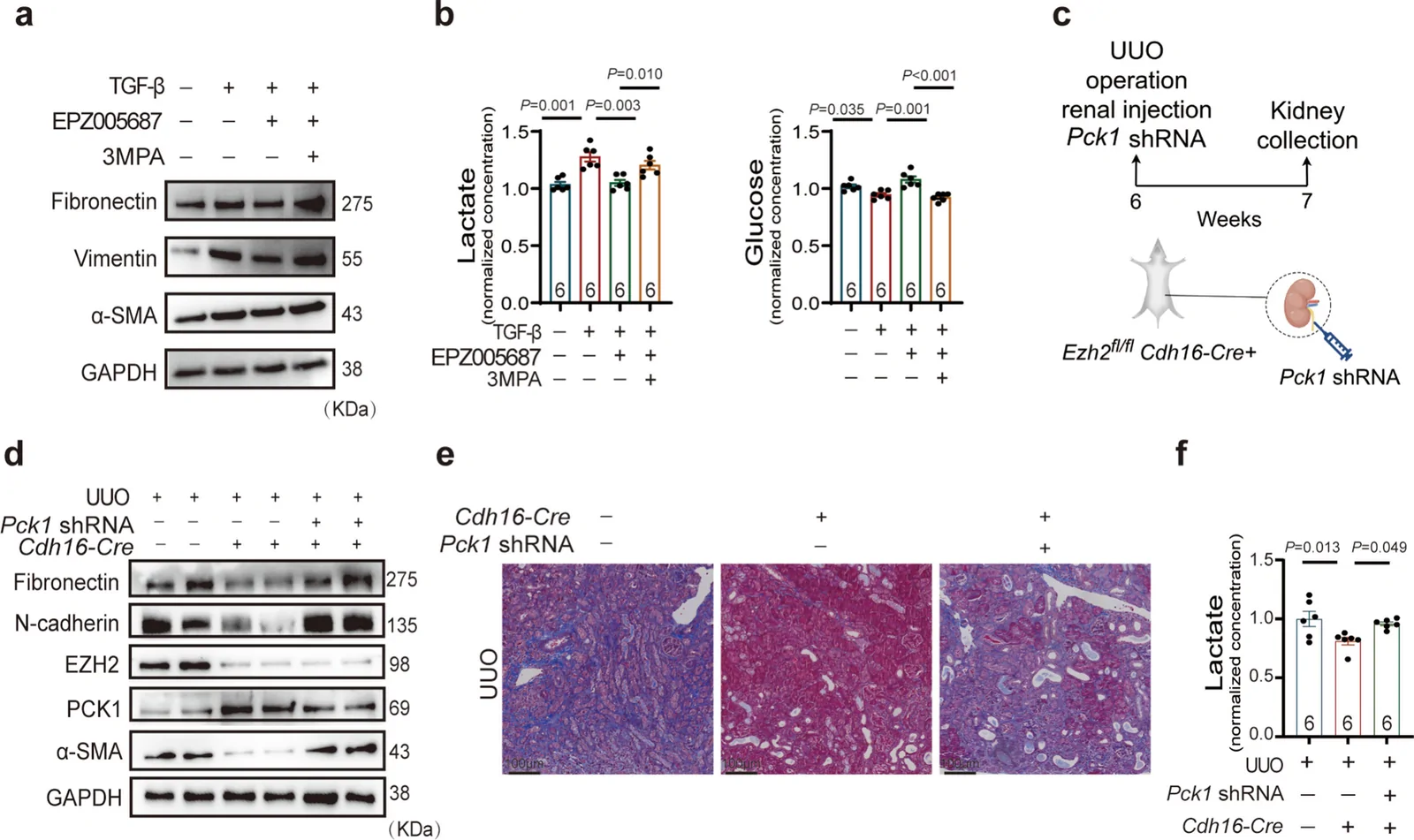
EZH2 promotes renal fibrosis through inhibition of renal gluconeogenesis. **a** Western blot analysis of fibronectin, α-SMA, and vimentin, in HK-2 cells stimulated with TGF-β and treated with EPZ005687 (5 µM) or 3-mercaptopicolinic acid (3MPA, 250 µM) for 48 h. *n* = 3 per group. **b** Glucose and lactate concentrations in culture medium from HK-2 cells stimulated with TGF-β and treated with EPZ005687 (5 µM) or 3MPA (250 µM) for 48 h were measured, and normalized to the control group (without EPZ005687 or 3MPA treatment). *n* = 6 per group. **c** Experimental design: Male mice with tubular specific deletion of *Ezh2* (*Ezh2*^fl/fl^;*Cdh16-Cre*) at 6 weeks of age were injected with control or *Pck1* shRNA, subjected to UUO surgery, and sacrificed after 1 week (*n* = 7 mice per group). **d** Western blot analysis of fibronectin, N-cadherin, α-SMA, PCK1, and EZH2 in kidney samples. *n* = 6 per group. **e** Masson’s trichrome staining of renal fibrosis. Scale bar: 100 µm. *n* = 6 per group. **f** Kidney lactate levels were measured and normalized to UUO kidneys without *Ezh2* deletion and *Pck1* knockdown. *n* = 6 per group. Comparisons among multiple groups were performed using one-way ANOVA followed by Tukey’s post-hoc test. Data are presented as mean ± standard deviation

To establish whether EZH2 promotes fibrosis through suppression of gluconeogenesis, a series of pharmacological rescue experiments was performed in TGF-β-stimulated HK-2 cells. Co-treatment with the PCK1 inhibitor 3-mercaptopicolinic acid (3MPA) effectively reversed the EPZ005687-induced downregulation of fibrotic markers (fibronectin, vimentin, and α-SMA) (Fig. 6a and Fig. S9a), as well as the EPZ005687-induced metabolic changes, including decreased lactate and increased glucose levels (Fig. 6b). Similarly, inhibition of FBP1, another rate-limiting enzyme in gluconeogenesis, using FBPase-1 inhibitor‑1 also abrogated the anti-fibrotic (reduced fibronectin, vimentin, and α-SMA expression) and metabolic effects (decreased lactate and increased glucose levels) of EPZ005687 (Fig. S9b-d). These results collectively indicate that the pro-fibrotic action of EZH2 is dependent on an intact gluconeogenic pathway. Because FBP1 operates downstream of PCK1, blockade at either step abolishes gluconeogenic output and thereby prevents the anti-fibrotic effect of EZH2 inhibition.

In vivo, *Pck1* shRNA nullified the antifibrotic effects of tubular *Ezh2* deletion, restoring fibrotic-marker (fibronectin and N-cadherin) expression, matrix deposition, and lactate accumulation (Fig. 6c-f, Fig. S9e, f). No significant differences were observed between groups in renal glucose levels (Fig. S9g). Thus, PCK1 is an essential downstream mediator of EZH2-driven fibrosis.

### Pharmacological inhibition of EZH2 promotes renal gluconeogenesis in CKD mouse models

**Fig. 7:**
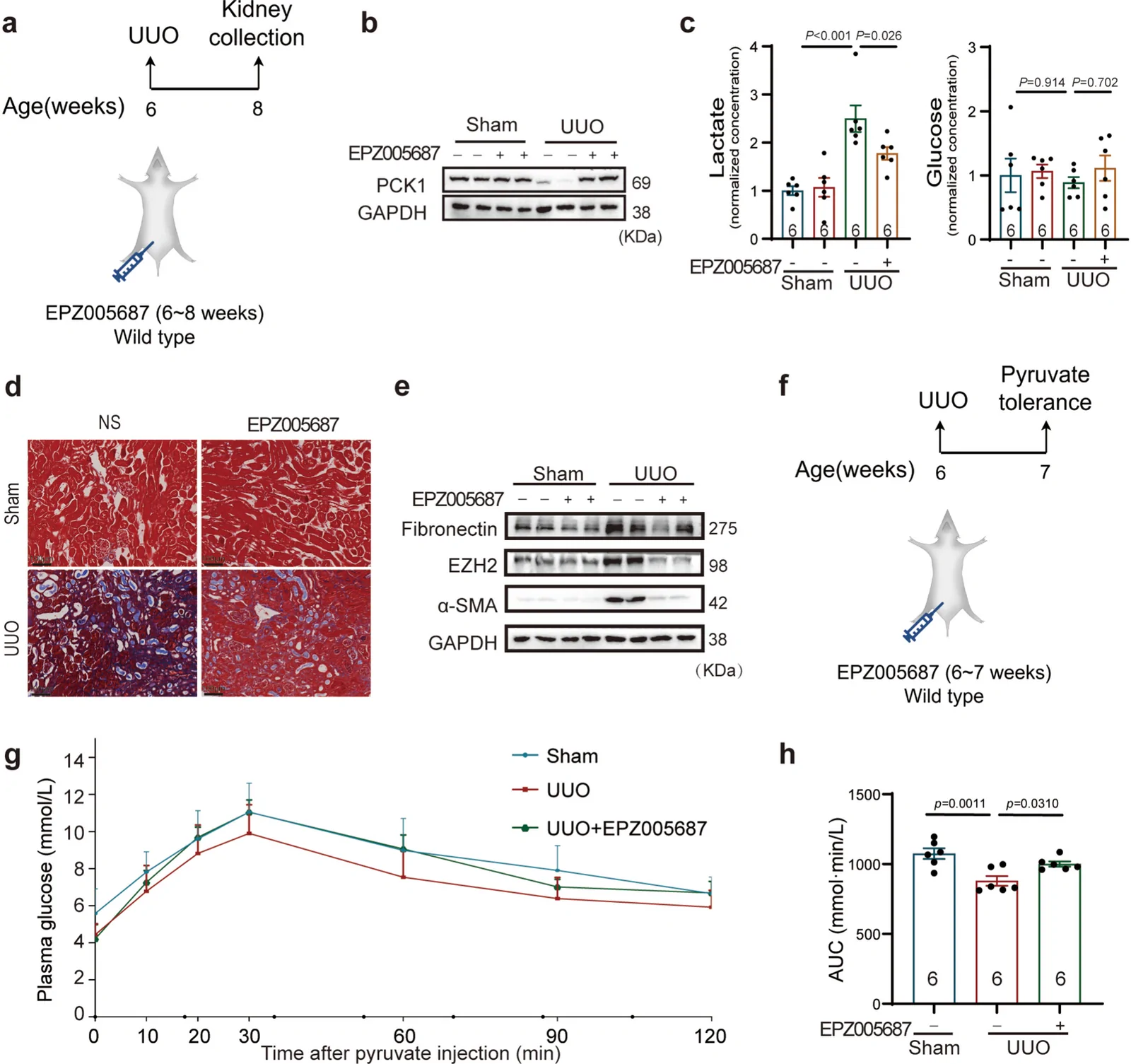
Pharmacological inhibition of EZH2 promotes renal gluconeogenesis in CKD mouse model. **a** Experimental design: Wild-type (WT) male mice at 6 weeks of age underwent sham or UUO surgery, which were followed by treatment with normal saline (NS) or EPZ005687 (10 mg/kg daily) for 14 days (*n* = 6 per group). **b** Western blot analysis of PCK1 expression in sham or UUO mouse kidneys after EZH2 inhibition. *n* = 6 per group. **c** Kidney glucose and lactate levels were measured and normalized to sham kidneys. *n* = 6 per group. **d** Masson’s trichrome staining of renal fibrosis. Scale bar: 100 μm. *n* = 6 per group. **e** Western blot analysis of fibronectin, α-SMA, and EZH2. *n* = 6 per group. **f** Metabolic tolerance testing protocol: in an independent cohort, WT male mice underwent sham or UUO surgery followed by NS or EPZ005687 (10 mg/kg daily) for 7 days from day 0, and a pyruvate tolerance test (PTT) was performed on day 7. *n* = 6 per group. **g**-**h** Blood glucose levels after pyruvate injection in mice were recorded and area under curve (AUC) was analyzed. *n* = 6 per group. Comparisons among multiple groups were performed using one-way ANOVA followed by Tukey’s post-hoc test. Data are presented as mean ± standard deviation

Having validated the EZH2–PCK1 regulatory axis, the therapeutic potential of pharmacological EZH2 inhibition was next assessed in CKD. Wild-type mice were subjected to UUO and administered vehicle or EPZ005687 (10 mg/kg) for 14 consecutive days (Fig. 7a). EPZ005687 treatment upregulated PCK1 expression, reduced renal lactate accumulation, and markedly attenuated extracellular matrix deposition and the expression of fibrotic markers (fibronectin and α-SMA) in UUO-injured kidneys (Fig. 7b-e and Fig. S10a-c). To explore whether EZH2 inhibition might exert systemic metabolic effects in the context of CKD, a pyruvate tolerance test (PTT) was performed as an exploratory functional readout in an independent cohort of mice treated for 7 days. UUO mice showed a reduced PTT area under the curve (AUC) compared to sham-operated controls, suggesting impaired gluconeogenic capacity (Fig. 7f-h). Treatment with EPZ005687 was associated with a significant increase in PTT-AUC, indicating a potential improvement in pyruvate-to-glucose conversion (Fig. 7g, h).

Collectively, these results suggest that pharmacological EZH2 inhibition confers renal benefits in CKD models, potentially through modulation of the EZH2**–**PCK1 axis.

## Discussion

In this study, we identified a novel role for EZH2 in regulating renal gluconeogenesis through epigenetic repression of PCK1 and demonstrated its impact on CKD progression. Although transcriptional control of gluconeogenesis by factors like PPARα, FoxO1, and PGC1α is well-established [9, 22], our findings showed that epigenetic mechanisms are likewise pivotal. Using a comprehensive in vitro, ex vivo, and in vivo approach, we found an inverse relationship between EZH2 expression and gluconeogenic genes in proximal tubules, the primary site of renal gluconeogenesis. CUT&Tag profiling revealed that EZH2, together with its associated H3K27me3 mark, accumulates at the promoters of key gluconeogenic enzymes, thereby silencing their transcription. This epigenetic blockage of gluconeogenic enzymes represents a previously unrecognized mechanism of metabolic dysregulation in kidney diseases.

Notably, EZH2 occupancy was not restricted to a single locus but extended to the promoters of *PCK1*, *FBP1*, and *G6PC*, and all three transcripts were de-repressed upon *Ezh2* deletion, indicating that EZH2 represses a gluconeogenic program rather than an individual enzyme. Because these enzymes act sequentially within one linear pathway, loss-of-function at any single node abolishes gluconeogenic output and cannot, in principle, apportion the relative contribution of each enzyme. Within this program, PCK1 is the node for which causal, bidirectional evidence was obtained both in vitro and in vivo, and it is therefore best regarded as the principal effector identified in the present study rather than as the exclusive mediator of the EZH2 effect.

Beyond direct transcriptional repression, EZH2 may also regulate gluconeogenesis indirectly through transcriptional factors such as FoxO1 and PGC1α. A recent study showed that EZH2 epigenetically silences the expression of *PGC1α* gene [23]. EZH2 is upstream of *FoxO1* and negatively regulates its expression [24].

Across multiple models of renal fibrosis, EZH2 inhibition consistently restored gluconeogenic gene expression and reduced lactate accumulation, underscoring its central role in metabolic control. Far from being a mere waste product of glycolysis, lactate has emerged as a key regulator of cellular signaling [25, 26]. Recent studies link lactate to kidney-disease progression via histone and non-histone lactylation [27–30]. Thus, EZH2 may influence disease not only through metabolic reprogramming but also by modulating lactylation pathways that warrant further investigation.

Despite marked changes in lactate metabolism, renal tissue glucose levels were not significantly altered in vivo, probably because glomerular filtration, tubular reabsorption, and overall kidney function jointly modulate tissue glucose levels; notably, ^13^C tracing nonetheless revealed increased labelling of hexose phosphate intermediates, indicating that steady-state tissue glucose is an insensitive surrogate for gluconeogenic flux. By contrast, our cell-based studies clearly demonstrated that fibrotic stimuli impair gluconeogenic output, an effect restored by EZH2 inhibition, highlighting the complexity of translating cellular metabolism to whole-organ measurements.

Our findings also assign a protective function to PCK1 in the kidney, supported by experimental knockdown or overexpression of PCK1 in renal cells or fibrotic kidneys. A similar protective effect has been observed in the liver, where PCK1 limits fibrosis and tumorigenesis [31–34]. However, this protection may not be confined to canonical gluconeogenesis; it could also involve PCK1’s kinase activity or other non-classical mechanisms [31]. In line with this notion, our in vitro experiments showed that fibrotic changes were disproportionately more pronounced than the relatively modest metabolic shifts (in glucose and lactate) induced by pharmacological or genetic modulation of EZH2 or PCK1. This discrepancy suggests that additional EZH2 target genes or non-canonical functions of PCK1 are likely implicated in the observed pro- or anti-fibrotic effects. Future studies are warranted to delineate these alternative pathways and their relative contributions.

PCK1 diverts carbon from the TCA cycle by promoting cataplerosis [35], whereas EZH2 enhances TCA flux [14], suggesting a complex metabolic interplay. Deciphering this crosstalk may uncover additional therapeutic targets for metabolic reprogramming in CKD. Our ^13^C-lactate tracing provides labelling-level support for this crosstalk, showing that *Ezh2* deletion increases the labelling fractions of hexose phosphate intermediates while reducing that of acetyl-CoA. These reciprocal changes suggest that restoration of PCK1 in *Ezh2*-deficient tubules redirects pyruvate away from pyruvate dehydrogenase-mediated oxidation towards phosphoenolpyruvate formation, thereby favoring gluconeogenic carbon utilization. Because labelling of downstream TCA cycle intermediates was largely preserved, our data are best interpreted as a shift in carbon partitioning at the pyruvate node rather than a global reduction in oxidative flux. This directional change is nonetheless consistent with enhanced PCK1-dependent cataplerosis, and agrees with the converse phenotype reported after tubular *Pck1* loss [36].

Impaired renal gluconeogenesis is an early event in CKD that worsens with disease progression [10]. Our findings suggest that enhancing PCK1-mediated gluconeogenesis mitigates renal fibrosis, supporting therapeutic strategies targeting this pathway. Currently, there is no selective drug available to boost renal gluconeogenesis, although SGLT2 inhibitors and steroids correlate with increased renal glucose production [8]. The present data indicate that pharmacological EZH2 inhibition may restore renal gluconeogenesis and attenuate fibrosis in preclinical models, suggesting that this pathway merits further therapeutic exploration pending rigorous safety evaluation.

While EZH2 inhibition is therefore an attractive strategy, its translation still requires careful evaluation of safety profiles and long-term efficacy. EZH2 inhibition has shown promise in kidney disease and oncology, with multiple inhibitors advancing to clinical trials [37, 38]. However, the recent withdrawal of tazemetostat (Tazverik®) on March 9, 2026, due to increased secondary hematologic malignancies in the SYMPHONY-1 trial, underscores significant safety concerns [39]. For chronic non-terminal conditions such as kidney disease, this risk profile demands even greater caution than in oncology. Mechanistic investigation into whether the hematologic toxicity is class-specific (affecting all PRC2 inhibitors) versus compound-specific remains essential. Therefore, therapeutic claims must be tempered until long-term safety profiles, off-target effects, and mechanistic risks are fully elucidated through comprehensive preclinical and clinical studies.

This study has several limitations. First, genome-wide EZH2 occupancy was mapped by CUT&Tag only in the immortalized HK-2 line, and in vivo confirmation was obtained at the locus level by CUT&Tag-qPCR in unsorted primary cortical cells rather than in purified proximal tubular cells; although the tubule-specific phenotype of *Ezh2*^*fl/fl*^*;Cdh16-Cre* mice and the snRNA-seq data are consistent with a tubule-intrinsic mechanism, targeted profiling in sorted proximal tubular nuclei is required for definitive proof. Second, ^13^C-lactate tracing was performed at a single time point and therefore defines the direction of isotopologue partitioning but not absolute flux rates; multi-time-point tracing with kinetic modelling will be required for absolute quantification. Third, the tracer reports carbon entry at the pyruvate node: the reduced acetyl-CoA labelling fraction suggests diminished pyruvate dehydrogenase-mediated oxidation, but the unchanged labelling of downstream TCA cycle intermediates indicates that our data cannot resolve the broader mitochondrial oxidative changes that may accompany PCK1 restoration. Fourth, systemic administration of the EZH2 inhibitor may affect multiple tissues, and without measurement of serum insulin or hepatic gluconeogenic enzyme expression (*Pck1*, *G6pc*, and *Fbp1*), the tolerance test results cannot be causally attributed to renal EZH2–PCK1 signaling. Future studies employing conditional renal tubular *Ezh2* deletion are warranted for definitive causal validation. Fifth, although EZH2 occupancy and H3K27me3 enrichment were detected at the *PCK1*, *FBP1* and *G6PC* promoters and all three transcripts were de-repressed upon *Ezh2* deletion, the functional evidence is not equivalent across these genes: bidirectional gain- and loss-of-function experiments were performed only for PCK1, FBP1 was interrogated pharmacologically only, and G6PC was not functionally tested, in part because no selective inhibitor of the G6PC catalytic subunit is currently available for cell-based studies. FBP1 was also selected for its pharmacological tractability rather than for the strength of the human correlation, as EZH2 correlated inversely with PCK1 and G6PC but not with FBP1 in the allograft cohort. The relative contribution of individual gluconeogenic enzymes, and the possible involvement of additional EZH2 target genes acting in parallel, therefore remain to be defined. Finally, all in vivo experiments used male mice to minimize hormonal variability; future studies in female animals are needed to confirm sex-independent effects.

In conclusion, EZH2-mediated repression of renal gluconeogenic enzymes, particularly PCK1, drives metabolic dysfunction and fibrosis in CKD. Pharmacological or genetic relief of this epigenetic suppression restores tubular metabolism and curbs fibrosis, identifying the EZH2-PCK1 axis as an attractive target for CKD therapy.

## Materials and methods

### Animals

Male wild-type C57BL/6 J mice were sourced from Shanghai Model Organisms Center. *Ezh2*^*fl/fl*^ mice were kindly provided by Prof. Xi Wang [40]. To generate experimental lines, *Ezh2*^*fl/fl*^ mice were bred to either systemic tamoxifen-inducible B6.Cg-Tg (*CAG-cre/Esr1*) 5Amc/J mice (Jackson Laboratory, stock no. 004682) [41] or *Cdh16-Cre* transgenic mice (Jackson Laboratory, stock no. 012237). Because the *Cdh16* (Ksp-cadherin) promoter drives *Cre* expression predominantly in renal tubular epithelium, including proximal tubular cells where gluconeogenesis is most active, this line enabled tubule-restricted deletion of *Ezh2* [42]. The efficiency of tubular *Ezh2* deletion mediated by *Cdh16-Cre* was validated through genotyping, Western blot analysis of EZH2 protein expression in primary renal tubules, and immunofluorescence co-localization of EZH2 with the tubular epithelial marker LRP2 (Fig. S11a-d). All procedures were approved by the local ethics committee (protocol PZSHUTCM18102604) and conformed to institutional guidelines for animal care and use.

### Animal models

#### Unilateral ureteral obstruction (UUO)

UUO was established in male mice (18–22 g) as previously described [43]. For inducible deletion of *Ezh2*, *Ezh2*^*fl/fl*^*;Cre/Esr1*^+^ mice received three intraperitoneal injections of tamoxifen (50 mg/kg) one week before surgery. The left ureter was ligated to generate UUO; sham mice underwent identical surgery without ligation. From the day of surgery, mice received daily intraperitoneal injections of vehicle (normal saline) or EPZ005687 (10 mg/kg, T1905, TargetMol, Shanghai, China). Animals were sacrificed 14 days after UUO, and kidneys were harvested for analysis.

#### Folic acid (FA) nephropathy

FA-induced nephropathy model was established according to a previously described protocol [44]. Briefly, male mice (25–30 g) were administered a single dose of FA (250 mg/kg; Sigma-Aldrich, F7876) via intraperitoneal injection, with the compound solubilized in 0.3 M sodium bicarbonate to induce kidney injury. Control mice received an equivalent volume of the sodium bicarbonate vehicle alone. For *Ezh2*-deletion studies, *Ezh2*^*fl/fl*^*;Cre/Esr1*^+^ mice were pre-treated with tamoxifen as described above.

### Intra-renal injection

Intra-renal adenoviral administration was performed via retrograde ureteral infusion as previously described [45]. Mice received 50 µL normal saline containing 0.04% trypan blue and 0.5 × 10^9^ pfu of either *PCK1*-overexpression adenovirus, *Pck1* shRNA, or empty vector control (Vigene Biosciences, Jinan, China). UUO surgery was performed immediately after adenoviral administration. Mice were sacrificed 7 days after UUO surgery.

### Cell culture

Human proximal tubular epithelial cells (HK-2; GNHu47, Shanghai Institute of Biological Sciences, China) were maintained in DMEM/F12 medium (11330032, Gibco, NY, USA) containing 10% fetal bovine serum (FBS; 04–001-1, Biological Industries) along with 100 U/mL penicillin and 100 μg/mL streptomycin, and incubated under standard conditions (37 °C, 5% CO_2_) in a Thermo Scientific™ incubator (Cat. No. 311, MA, USA). For TGF-β stimulation, HK-2 cells were seeded in 6-well plates (3516, Corning Life Sciences, Suzhou, China). Sub-confluent cells (70%–80%) were serum-starved (0.5% FBS) for 12 h and then treated with TGF-β (2.5 ng/mL; 100–21, Peprotech, NJ, USA) for different time periods according to the experimental design.

For chemical inhibition studies, cells were treated with EPZ005687 (5 μM; T1905, TargetMol, Shanghai, China), FBP1 inhibitor FBPase-1 inhibitor-1 (1 µM; T22081, TargetMol, Shanghai, China), or the PCK1 inhibitor 3-mercaptopicolinic acid (3MPA; 250 µM; T12929, TargetMol, Shanghai, China) concurrently with TGF-β addition.

For adenoviral transfection, HK-2 cells or primary cells at ~ 30% confluence were infected with adenovirus encoding *EZH2* (Ad-*EZH2*), *PCK1* (Ad-*PCK1*), *Pck1* shRNA, or their respective controls at a multiplicity of infection (MOI) of 10. Overexpression vectors were constructed in the pADM-FH-GFP vector, whereas the shRNA vector used pADM-U6-GFP (Vigene Biosciences, Jinan, China). Medium was replaced 4 h after infection, and cells were stimulated with TGF-β 24 h later. Conditioned medium, RNA, and protein were collected at 24 or 48 h after TGF-β stimulation as indicated.

### Preparation and in vitro expansion of primary renal tubules and tubular epithelial cells

Primary proximal tubules were isolated from mouse kidneys as described [46]. Briefly, renal cortices were dissected, minced, and digested with collagenase II under mild agitation at 37 °C for 10 min. The supernatant was chilled on ice to precipitate tubules. For ex vivo assays, freshly isolated tubules were seeded in 12-well plates (3513, Corning Life Sciences, Suzhou, China) containing phenol-red-free DMEM/F12.

For primary cultures, outgrowing tubular cells were washed and given fresh medium after 48 h. Cells were harvested at 50% confluence and replated in 6-well plates for experiments.

### Single-nucleus RNA-seq analysis

Our single-nucleus RNA sequencing (snRNA-seq) datasets generated from the bilateral ischemia–reperfusion injury (IRI) model and reported previously were re-analyzed [47–49]. In one dataset, male C57BL/6 J mice aged 8–10 weeks underwent 18.5-min pedicle clamping, and kidneys were collected at multiple time points following IRI: 4 h, 12 h, 48 h, 14 days, and 6 weeks [49]. A second dataset used 15-min pedicle clamping with sampling at 64 h, 96 h, and 28 days [47]. Untreated kidneys processed identically served as controls [48]. Raw and processed data have been archived in the NCBI GEO database (GSE163863, GSE139107, and GSE151167).

Data processing was performed in Seurat (v 4.0.4) under R (v 4.3.1). After batch-effect correction, 64,316 nuclei from 33 samples were retained. Standard workflows (ScaleData, RunPCA, FindNeighbors, FindClusters; resolution = 0.5) were applied. Clusters were reassigned based on lineage-specific markers and time points. Integration was conducted in R v4.2.1, with box plots generated in R v4.3.1.

### Human data analysis

In this study, a secondary analysis was performed on a publicly available transcriptomic dataset originally generated by Cippà et al. (GEO accession: GSE126805) [50]. The original dataset comprises kidney allograft protocol biopsies from 41 kidney transplant recipients treated at the University Hospitals of Leuven. Protocol biopsies were collected at four time points: pre-implantation, post-reperfusion, 3 months, and 12 months post-transplant. The AKI to CKD transition was characterized by pseudotime analysis of the 3- and 12-month samples [50]. The resulting trajectory bifurcated into two branches: one designated as “early transition to CKD” and “CKD” (progression to fibrosis), and the other as “recovery”. This research adheres to the ethical standards outlined in the 2013 revision of the Declaration of Helsinki (World Medical Association) and was approved by the Ethics Committee of the University Hospitals of Leuven (approval nos. S53364 and S59572).

### RNA-seq analysis

Total RNA was isolated from UUO kidneys of control and *Ezh2* knockout mice (*n* = 3 per group) 14 days after surgery using TRIzol (15596026CN, Life Technologies, CA, USA). RNA integrity was evaluated using an Agilent 2100 Bioanalyzer, and only samples meeting the quality threshold (RIN > 8) proceeded to downstream analysis. Libraries were constructed by Biomarker Cloud Technologies (Beijing, China) and subjected to paired-end sequencing (150 bp) on an Illumina NovaSeq instrument. After trimming of adaptors and filtering out low-quality sequences, high-quality reads were mapped against the mouse reference genome (mm10) with Hisat2 (v2.2.1). Differential expression was determined with the DESeq2_EBSeq pipeline (false discovery rate (FDR) ≤ 0.05 and absolute fold-change > 2). Functional enrichment was carried out using KOBAS 3.0. Raw data have been archived in the NCBI BioProject repository (PRJNA793768).

### CUT&Tag

TGF-β-stimulated HK-2 cells were cryopreserved (10% DMSO, 20% FBS) and shipped to Biomarker Technologies for Cleavage Under Targets and Tagmentation (CUT&Tag) library construction. For each assay, 6 × 10^4^ to 1 × 10^5^ cells were bound to concanavalin A magnetic beads and incubated with rabbit anti-EZH2 antibody (5246, CST, MA, USA) or rabbit IgG (3900, CST, MA, USA). Hyperactive Tn5 transposase was used to tagment DNA proximal to antibody-bound chromatin. Libraries were sequenced on an Illumina NovaSeq 6000. Reads were trimmed with Cutadapt and aligned to hg38 with Bowtie2. Peaks were called with MACS2, annotated with ChIPseeker, and differential peaks were identified with DiffBind. Data are available in BioProject (PRJNA797258).

### Gluconeogenic intermediates measurements

Renal cortex was homogenized in 10 × normal saline. Lactate and glucose concentrations were measured with commercial kits (A019-2-1 and F006-1-1, Nanjing Jiancheng, Jiangsu, China) at 530 nm and 505 nm, respectively, and normalized to protein content. Culture supernatants were cleared by centrifugation before analysis.

### Metabolic tolerance test

Control or CKD mice received daily intraperitoneal injections of normal saline (NS) or EPZ005687 (10 mg/kg, T1905, TargetMol, Shanghai, China) for 7 days. Pyruvate tolerance tests were conducted on day 7 in a cohort independent of the one used for the 14-day histological and biochemical analysis. Mice were fasted for 16 h before the test. Tail blood glucose was monitored with an Accu-Chek glucometer (Roche, Basel, Switzerland) at baseline and at multiple time points (10, 20, 30, 60, 90, and 120 min) following intraperitoneal administration of sodium pyruvate (2 g/kg; A501259, Sangon Biotech, Shanghai, China). Areas under the curve (AUCs) were calculated by the trapezoidal rule in GraphPad Prism (GraphPad Software, MA, USA).

### Metabolic flux analysis by ^13^C-lactate tracing

To evaluate renal gluconeogenic carbon partitioning in vivo, stable isotope tracing was performed using L-Lactic acid-^13^C₃ sodium (TMID-0512, TargetMol, Shanghai, China; CAS: 201595-71-3, 98% isotopic enrichment), a ^13^C-labeled tracer specifically designed for lactate metabolism studies. Male mice with or without tubular-specific deletion of *Ezh2* (*n* = 6 per group) underwent UUO. On day 7 after UUO, following a 16-h fast, the tracer was administered intraperitoneally at a total dose of 10 mmol/kg in three equal injections at 15-min intervals. Renal cortex tissues were collected 60 min after the first injection and processed for LC–MS/MS analysis.

LC–MS was performed on a Thermo QE-HF-X using a Waters BEH Amide column (2.1 mm × 10 cm). Mobile phase A: water + 25 mM ammonium acetate + 25 mM ammonia; Phase B: acetonitrile. Gradient: 95% B (0–0.5 min), linear to 65% B (7 min), to 40% B (8 min), back to 95% B (9.1 min). Flow rate: 0.5 ml/min. MS settings: tSIM mode (positive and negative separately), resolution 120,000, AGC target 3e6, max IT 200 ms, spray voltage 3500/4000 V, capillary temperature 320 °C. Data processing: Skyline and MAVEN for peak extraction; R package accucor for natural abundance correction (resolution 60,000), yielding Abundance (corrected intensity) and Fraction/MID (isotopologue distribution) matrices. Isotopologue data are reported both as abundances and as fractional labelling (the proportion of each isotopologue relative to the total metabolite pool). Fractional labelling is presented as the primary readout because it accounts for genotype-related differences in metabolite pool sizes, whereas abundance data are provided as supporting evidence. Reproducibility was verified by PCA and Pearson correlation (*r* > 0.8). Differential analysis was performed using a t-test with false discovery rate correction by the Benjamini–Hochberg method, with thresholds of *p* < 0.05 and |log_2_FC|> 0.5.

### Statistical analysis

Data are presented as mean ± standard deviation (SD). Multiple groups were compared by one-way analysis of variance (ANOVA) with Tukey’s post-hoc test; two-group comparisons used unpaired two-tailed Student’s *t*-tests. *EZH2* expression among human transcriptomic groups was analyzed by Kruskal–Wallis test with Dunn’s correction. Pearson correlation assessed linear relationships between *EZH2* and gluconeogenic-gene expression in CKD patient kidneys (*n* = 163). All analyses were performed using GraphPad Prism v8.0, with statistical significance defined as *p* < 0.05.

## Supplementary Information

Supplementary Material 1.

## Data Availability

RNA-seq data from UUO kidneys are deposited in NCBI BioProject (PRJNA793768); CUT&Tag sequencing data are archived in NCBI BioProject (PRJNA797258); snRNA-seq datasets are available in NCBI GEO (GSE163863, GSE139107, GSE151167). The human kidney allograft transcriptomic dataset analyzed in this study was originally generated by Cippà et al. and is publicly available in NCBI GEO under accession number GSE126805.
