# Supplementary material for: Epigenetically silencing gluconeogenic enzyme PCK1 by EZH2 promotes renal tubulointerstitial fibrosis
Source: Mol Biomed. 2026 Oct 14;7:210. doi: 10.1186/s43556-026-00617-y (PMC13669293; doi:10.1186/s43556-026-00617-y)
Supplement: Supplementary file 1 [file 43556_2026_617_MOESM1_ESM.docx]

**Supplementary Information**

**Epigenetically silencing gluconeogenic enzyme PCK1 by EZH2 promotes renal tubulointerstitial fibrosis**

Ming Wu^1,2,3,*,†^, Pinglan Lin^1,2,3,†^, Zhiqiang Yan^4,7,†^, Yanzhe Wang ^1,2,3^, Anna Rinaldi^5,6^, Pietro E Cippà^5, 6^, Di Huang^1,2,3^, Jiayi Qiao^4,7^, Aidong Yang^7^, Bo Tan^4,7, *^, Chaoyang Ye^1,2,3, *^, Dongping Chen^1,2,3, *^

^1^Department of Nephrology, Shuguang Hospital Affiliated to Shanghai University of Traditional Chinese Medicine, Shanghai, China.

^2^TCM Institute of Kidney Disease of Shanghai University of Traditional Chinese Medicine, Shanghai, China.

^3^Key Laboratory of Liver and Kidney Diseases, Ministry of Education, Shanghai Key Laboratory of Traditional Chinese Clinical Medicine, Shanghai, China.

^4^Clinical Pharmacokinetic Laboratory, Shuguang Hospital Affiliated to Shanghai University of Traditional Chinese Medicine, Shanghai, China.

^5^Department of Medicine, Division of Nephrology, Ente Ospedaliero Cantonale, Lugano, Switzerland.

^6^Laboratories for Translational Research, Ente Ospedaliero Cantonale, Bellinzona, Switzerland.

^7^The Research Center for Traditional Chinese Medicine, Shanghai Institute of Infectious Diseases and Biosecurity, School of Traditional Chinese Medicine, Shanghai University of Traditional Chinese Medicine, Shanghai, China.

†These authors contributed equally to this paper.

*Corresponding Author 1: Dongping Chen, E-mail: 13764362569@163.com

*Corresponding Author 2: Chaoyang Ye, E-mail: yechaoyang63@126.com

*Corresponding Author 3: Ming Wu, E-mail: mingwunl@126.com

*Corresponding Author 4: Bo Tan, E-mail: tbot@163.com

**Supplementary Methods**

**Genotyping**

Mouse genomic DNA was extracted from tail tips using the Animal Genomic DNA Quick Extraction Kit for PCR Analysis (D0065, Beyotime Biotechnology, Shanghai, China). For *Ezh2* genotyping, primers *Ezh2*-F (5′-CCCATGTTTAAGGGCATAGTGACATG-3′) and *Ezh2*-R (5′-ATGTGCAGGTCAGTCAGCAACTTCAG-3′) were used, and Cre recombination was detected using *Cdh16-Cre*-F (5′-GCAGATCTGGCTCTCCAAAG-3′) and *Cdh16-Cre*-R (5′-AGGCAAATTTTGGTGTACGG-3′). PCR reactions were performed with 2× Premix Taq (AG11109-S, Accurate Biotechnology, Changsha, Hunan, China), and products were resolved on agarose gels stained with GelRed, visualized under UV light, and sized against a DNA ladder.

**Quantitative PCR (qPCR)**

Total RNA from either kidney tissue or renal cells was extracted with TRIzol (R401-01, Vazyme, Jiangsu, China) and reverse-transcribed using the PrimeScript RT reagent kit (RR0036A, Takara). qPCR was performed with Hieff Unicon^®^ Probe Master Mix (11205E, Yeasen, Shanghai, China) on a StepOnePlus System (Applied Biosystems, CA, USA). Thermocycling parameters: initial denaturation: 95°C, 5 min. PCR amplification (40 cycles): 95°C, 10 s; 60°C, 30 s. Melt curve analysis: 95°C, 15 s; 60°C, 1 min; 95°C, 15 s. Primer sequences are listed in Table S1.

**CUT&Tag-qPCR validation**

CUT&Tag-qPCR was performed on HK-2 cells and renal primary cells. Primary single cells were isolated from renal cortex tissue according to the manufacturer’s instructions (Stemcell Technologies, BC, Canada). In brief, renal cortex tissues from mice were finely minced and dissociated for 2 hours in a gentle collagenase/hyaluronidase solution (07912, Stemcell Technologies, BC, Canada), diluted in DMEM/F12 with 15 mM HEPES and supplemented with 2% (w/v) Fraction V BSA. A single-cell suspension was then obtained by further digesting the tissue with 0.05% trypsin-EDTA (C0204, Beyotime, Jiangsu, China) and 1 U/mL dispase (07923, Stemcell Technologies, BC, Canada). Finally, cell clumps were removed by filtration through a 40-μm cell strainer.

Targeted CUT&Tag experiment was performed with the Hyperactive In-Situ ChIP Kit (TD901, Vazyme, Jiangsu, China) using antibodies against EZH2 (5246, CST, MA, USA), H3K27me3 (9733, CST, MA, USA), or rabbit IgG (3900, CST, MA, USA). Promoter enrichment for human or mouse genes was quantified by qPCR (primers in Table S2) and normalized to input DNA and IgG controls.

**Masson’s trichrome** **staining**

Paraffin-embedded kidney sections (4 μm) were dewaxed, rehydrated, and stained with Masson’s trichrome. Images were captured on a Nikon 80i microscope. For morphometry, 10 random cortical fields (×400) per kidney were analyzed with ImageJ by an investigator blinded to group allocation. Fibrosis was expressed as the percentage of blue-stained area.

**Immunofluorescence**

Paraffin-embedded kidney sections (4 µm) underwent antigen retrieval in EDTA (pH 9.0), blocking in 5% BSA, and overnight incubation at 4°C with primary antibodies against EZH2 (1:100; 5246, CST, MA, USA) and LRP2 (1:100; A3612, Abclonal, Hubei, China), followed by incubation with a secondary antibody conjugated to horseradish peroxidase (HRP) (1:2000; RCA054, Huilanbio, Shanghai, China). A three-color fluorescence kit based on the tyramide signal amplification (TSA) technology (RC0086-23RM, Recordbio Biological Technology, shanghai, China) was used for signal detection. Nuclei were visualized using DAPI-Fluoromount G (0100-20, Southern Biotech, AL, USA). Images were acquired through a scanner (HL-MIC, Huilanbio, Shanghai, China) and analyzed by image analysis software (HL-Merge, Huilanbio, Shanghai, China).

**Western blotting**

Proteins from kidney tissue, cultured cells, or isolated tubules were extracted in RIPA buffer (P0013B, Beyotime, Jiangsu, China), separated on 8-10% SDS-PAGE, and transferred to PVDF membranes. After 5% milk blocking for 1 h at room temperature, membranes were probed with the following primary antibodies (1:1000 unless stated) during an overnight incubation at 4°C: Fibronectin (ab23750, Abcam, Cambridge, UK), collagen-I (1:500; sc-293182, Santa Cruz, CA, USA), α-SMA (ET1607-53, HUABIO, Zhejiang, China), EZH2 (5246, CST, MA, USA), PCK1 (144-02036, Raybiotech, GA, USA), GAPDH (1:5000; 60004-1-Ig, Proteintech, IL, USA), N-cadherin (sc-59987, Santa Cruz, CA, USA), FBP1 (ET7110-07, HUABIO, Zhejiang, China), or α-tubulin (AF0001, Beyotime, Jiangsu, China). Bands were visualized by ECL (P0018A, Beyotime, Jiangsu, China) after incubation with HRP-linked secondary antibodies (1:1000; A0216 or A0208, Beyotime, Jiangsu, China) for 1 h at room temperature and quantified in ImageJ.

**Supplementary Figures**

**Fig. S1** EZH2 promotes renal fibrosis in injured kidneys.

Quantification of western blot and Masson's trichrome staining data from Figure 1 and Figure 2. (a) Male wild‑type (WT) mice were subjected to sham surgery or unilateral ureteral obstruction (UUO) and sacrificed 14 days post‑operation (n = 7 per group). Densitometric quantification of western blot analysis for fibronectin, N‑cadherin, EZH2, and α‑SMA expression in kidney tissues. n = 6 per group. (b) Renal fibrosis evaluation by Masson’s trichrome staining in control (*Ezh2^fl/fl^;Cre/Esr1^-^*) and *Ezh2*-deleted (*Ezh2^fl/fl^;Cre/Esr1^+^*) mice after Sham or UUO surgery, with corresponding quantification. n = 6 per group. (c) Densitometric quantification of western blot analysis for fibronectin, collagen-I, α-SMA, EZH2, and PCK1 expression in renal tissues. n = 6 per group. In animal experiments, each data point corresponded to one animal. Comparisons among multiple groups were performed using one-way ANOVA followed by Tukey’s post-hoc test, while comparisons between two groups were conducted using unpaired two-tailed Student’s *t*‑tests. Data are presented as mean ± standard deviation.

**Figure S2.** TGF-β promotes EZH2 expression in renal epithelial cells.

Quantification for Western blot analysis of fibronectin, vimentin, EZH2, and α‑SMA in HK-2 cells stimulated with TGF-β at different time points. n = 3 per group. All cell‑based experiments were repeated independently three times, with each data point representing an independent biological sample. Comparisons among multiple groups were performed using one-way ANOVA followed by Tukey’s post-hoc test, while comparisons between two groups were conducted using unpaired two-tailed Student’s *t*‑tests. Data are presented as mean ± standard deviation.

**Fig. S3** Ablation of *Ezh2* increases PCK1 expression and inhibits renal fibrosis in folic acid-induced nephropathy.

Control (*Ezh2^f^*^l/fl^;*Cre/Esr1*^-^) and (*Ezh2*^fl/fl^;*Cre/Esr1*^+^) *Ezh2* conditional knockout male mice were injected with vehicle or FA intraperitoneally and were sacrificed on day 7. (a-c) The expression of fibronectin, collagen-I, α-SMA, EZH2 and PCK1 was analyzed by Western blotting. n = 6 per group. (d-e) Renal fibrosis was assessed by Masson’s trichrome staining and quantified. Scale bar represents 100 μm. n = 6 per group. In animal studies, each data point corresponded to one mouse. Comparisons among multiple groups were performed using one-way ANOVA followed by Tukey’s post-hoc test. Data are presented as mean ± standard deviation.

**Fig. S4** Inhibition of EZH2 attenuates fibrosis in TGF-β stimulated HK2 cells.

(a-b) HK2 human renal epithelial cells were starved overnight and followed by stimulation with TGF-β and treatment with 5 µM of EPZ005687 for 48 h. Cell lysates were extracted. The expression of fibronectin, α-SMA and EZH2 were analyzed by Western blotting and then quantified. n = 6 per group. All cell‑based experiments were repeated independently three times, with each data point representing an independent biological sample. Comparisons among multiple groups were performed using one-way ANOVA followed by Tukey’s post-hoc test. Data are presented as mean ± standard deviation.

**Fig. S5** Over-expression of *EZH2* promotes fibrosis in TGF-β stimulated HK2 cells.

(a-b) HK2 human renal epithelial cells were transfected with adenovirus encoding *EZH2* (Ad-*EZH2*) or control virus, and in the next day were stimulated with TGF-β in starvation medium for 48 h. The expression of fibronectin and EZH2 were analyzed by Western blotting and then quantified. n = 6 per group. All cell‑based experiments were repeated independently three times, with each data point representing an independent biological sample. Comparisons among multiple groups were performed using one-way ANOVA followed by Tukey’s post-hoc test. Data are presented as mean ± standard deviation.

**Fig. S6** Tubular deletion of *Ezh2* attenuates fibrosis in UUO mice.

Quantification of western blot and Masson's trichrome staining data from Figure 4. Male mice with tubular specific deletion of *Ezh2* (*Ezh2*^fl/fl^;*Cdh16-Cre*) were generated, and received sham or UUO operation at week 6 of age, which were sacrificed after two weeks (n = 7 mice per group). (a) Densitometric quantification of western blot analysis of PCK1 and EZH2 in kidney samples. n = 6 per group. (b) Quantification of Masson’s trichrome staining of renal fibrosis. n = 6 per group. (c-d) The expression of fibronectin, N-cadherin, and α-SMA were analyzed by Western blotting and further quantified. n = 6 per group. One representative of at least three independent experiments is shown. (e) Isotopologue abundances of ¹³C-lactate–derived metabolites in *Ezh2*-deficient kidneys. Male mice with tubular-specific deletion of *Ezh2* (*Ezh2*^fl/fl^;*Cdh16-Cre*) (referred to *Ezh2*_KO) or control (referred to *Ezh2*_WT) littermates were subjected to UUO. On day 7, mice received intraperitoneal ¹³C₃-lactate (10 mmol/kg in three divided doses) after a 16-h fast. Renal cortex was collected 60 min after the first injection. Natural-abundance–corrected isotopologue abundances of lactate (M+3), acetyl-CoA (M+2), fructose 6-phosphate (M+1) and glucose 6-phosphate (M+1) are shown. n = 6 per group. In animal studies, each data point corresponded to one mouse. Comparisons between two groups were conducted using unpaired two-tailed Student's t-tests with FDR correction (BH method). Data are presented as mean ± standard deviation. Comparisons among multiple groups were performed using one-way ANOVA followed by Tukey’s post-hoc test.

**Fig. S7** PCK1 knockdown or overexpression does not affect renal glucose levels in mice.

Quantification of western blot and Masson's trichrome staining data from Figure 5. (a) Quantification of western blot analysis of fibronectin, N-cadherin, vimentin, and PCK1 in primary tubular cells transfected with adenovirus encoding *PCK1* (Ad-PCK1) or control virus (Ad-Ctr) and stimulated with TGF-β for 48 h. n = 6 per group. (b) Quantification of western blot analysis of fibronectin, N-cadherin, vimentin, and PCK1 in primary tubular cells transfected with *Pck1* shRNA or control shRNA and stimulated with TGF-β for 48 h. n = 6 per group. (c) Quantification of western blot analysis and quantification of fibronectin, N-cadherin, and PCK1. n = 6 per group. (d) Quantification of Masson’s trichrome staining of renal fibrosis in UUO kidneys with control virus or Ad-*PCK1*. n = 6 per group. (e) Kidney glucose levels were measured and normalized to UUO kidneys without *PCK1* overexpression. n = 6 per group. (f) Quantification of western blot analysis and quantification of fibronectin, N-cadherin, and PCK1. n = 6 per group. (g) Quantification of Masson’s trichrome staining of renal fibrosis in UUO kidneys with control shRNA or *Pck1* shRNA. n = 6 per group. (h) Kidney glucose levels were measured and normalized to UUO kidneys without *Pck1* shRNA. n = 6 per group. In animal experiments, each data point corresponded to one animal. Comparisons between two groups were conducted using unpaired two-tailed Student’s *t*‑tests. Data are presented as mean ± standard deviation.

**Fig. S8** Inhibition of FBP1 promotes fibrotic responses in TGF-β stimulated HK2 cells.

(a-b) Western blot analysis and quantification of fibronectin, N-cadherin, vimentin, and FBP1 in HK-2 cells stimulated with TGF-β and treated with FBP1 inhibitor FBPase-1 inhibitor-1 (1 µM) for 48 h. n = 3 per group. (c) Glucose and lactate concentrations in the medium were measured, and normalized to the control group (without TGF‑β stimulation). n = 6 per group. All cell‑based experiments were repeated independently three times, with each data point representing an independent biological sample. Comparisons among multiple groups were performed using one-way ANOVA followed by Tukey’s post-hoc test. Data are presented as mean ± standard deviation.

**Fig. S9** EZH2 promotes renal fibrosis through inhibition of gluconeogenesis.

Quantification of western blot and Masson's trichrome staining data from Figure 6. (a) Quantification of western blot analysis of fibronectin, vimentin, and α-SMA, in HK-2 cells stimulated with TGF-β and treated with EPZ005687 (5 µM) or 3-mercaptopicolinic acid (3MPA, 250 µM) for 48 h. n = 3 per group. (b-c) Western blot analysis and quantification of western blot analysis of fibronectin, α-SMA, and vimentin, in HK-2 cells stimulated with TGF-β and treated with EPZ005687 (5 µM) or FBP1 inhibitor FBPase-1 inhibitor-1 (1 µM) for 48 h. n = 3 per group. (d) Glucose and lactate concentrations in the medium were measured, and normalized to the control group (without TGF‑β stimulation). n = 6 per group. (e) Quantification of western blot analysis of fibronectin, N-cadherin, α-SMA, PCK1, and EZH2 in kidney samples. n = 6 per group. (f) Quantification of Masson's trichrome staining of renal fibrosis. n = 6 per group. (g) Kidney glucose levels were measured and normalized to UUO kidneys without *Ezh2* deletion and *Pck1* knockdown. In animal experiments, each data point corresponded to one animal. Comparisons among multiple groups were performed using one-way ANOVA followed by Tukey’s post-hoc test.

**Fig. S10** Pharmacological inhibition of EZH2 promotes renal gluconeogenesis in CKD mouse model.

(a) Quantification of western blot analysis of PCK1 expression in sham or UUO male mouse kidneys after EZH2 inhibition. n = 6 per group. (b) Quantification of Masson's trichrome staining of renal fibrosis. n = 6 per group. (c) Quantification of western blot analysis of fibronectin, α-SMA, and EZH2. n = 6 per group. Comparisons among multiple groups were performed using one-way ANOVA followed by Tukey’s post-hoc test. Data are presented as mean ± standard deviation.

**Fig. S11** Tubular-specific deletion of *Ezh2* reduces EZH2 expression in proximal tubules.

(a) Mouse tail snips were collected, and genomic DNA was extracted for genotyping by PCR to detect the *Ezh2* floxed allele (around 300 bp) and *Cre* transgene (around 450 bp). (b) Primary kidney tubules were isolated from male mice with or without tubular-specific deletion of *Ezh2* (*Ezh2*^fl/fl^;*Cdh16-Cre*). The expression of EZH2 was analyzed by Western blotting and further quantified. n = 6 per group. (c) Immunofluorescence staining for EZH2 (green) and the proximal tubular marker LRP2 (purple) in UUO kidneys with or without tubular-specific deletion of *Ezh2* (*Ezh2*^fl/fl^;*Cdh16-Cre*). Nuclei were counterstained with DAPI (blue). Scale bars: 20 µm. n = 6 per group. (d) Quantification of EZH2 positive cells in proximal tubular cells (LRP2 positivity). n = 6 per group. In animal studies, each data point corresponded to one mouse. Comparisons between two groups were conducted using unpaired two-tailed Student’s t‑tests. Data are presented as mean ± standard deviation.

**Supplementary Tables**

Table S1: List of primers used for quantitative PCR

| Primer  Name | Forward | Reverse |
| --- | --- | --- |
| mouse  *Sgk2* | GAGACCACGTCCACCTTCTG | CGTAAGGCTCTTTACGAAGCA |
| mouse  *Gys2* | CAGCACCATTAGACGAATCGGAC | CCAAGGTGACAACCTCGGACAA |
| mouse  *Fgf1* | GTAGTTTCCTAGAGGCAGGTTG | TGATAAAGTGGAGTGAAGAGAGC |
| mouse  *Pck1* | CTCAGCTGGCAGCATGGGGTG | AACAGCTCCTCCACGTTGACG |
| mouse  *G6pc* | CCACTTTGGTTTCAGTTGAATCAG | GTATCCACCAGTAAGGACGATGG |
| mouse  *Fbp1* | GCTCTGCACCGCGATCA | ACATTGGTTGAGCCAGCGATA |
| mouse  *Tgf-β* | ACAACTGCTCCACCTTGGGCTTG | ACCATGCCAACTTCTGTCTGGGAC |
| mouse  *Gapdh* | AGGTCGGTGTGAACGGATTTG | TGTAGACCATGTAGTTGAGGTCA |
| human  *PCK1* | GGTTCCCAGGGTGCATGAAA | CACGTAGGGTGAATCCGTCAG |
| human  *GAPDH* | GAGTCAACGGATTTGGTCGT | TTGATTTTGGAGGGATCTCG |

Table S2: List of primers used for CUT&Tag-PCR

| Primer  Name | Forward | Reverse |
| --- | --- | --- |
| human  *PCK1* | GGCTGAAAGGAAGCCTGTGA | CAATGTTCCGCTCACCTTTCAT |
| human  *FBP1* | AGCGTCTTTCAGCCACTCTT | CAGCCTCACAATTGGGGTCA |
| human  *G6PC* | AGGTGGAACAGTTATGGCA | TGTCTTTGCTTACCATGGCCT |
| mouse  *Pck1* | AGTTTGCATCAGCAACAGGC | CCTCTGACGTAAGGGGCAG |
| mouse  *Fbp1* | TCCTTGGGCACTGTCTACCT | ACAGGAGCCACTTTGGCATA |
| mouse  *G6pc* | ACAAACACTCTCCGGACTGG | CTGCAGCAAAAAGCCACCAA |
